# Paediatric renal tumors

**DOI:** 10.1186/1470-7330-15-S1-O37

**Published:** 2015-10-02

**Authors:** Alexander J Towbin

**Affiliations:** 1 Cincinnati Children's Hospital, Department of Radiology, Cincinnati, Ohio 45229, USA

## 

Pediatric renal masses are uncommon and the differential diagnosis list is made up of a mix of benign and malignant lesions. While there is some overlap in the entities affecting children and adults, the majority of lesions arise in childhood. The child's age, clinical history, and imaging findings all help to distinguish the majority of tumors. The purpose of this interactive session is to review the renal lesions that affect the pediatric population, describe their imaging findings, and discuss how the different lesions are differentiated on imaging. The following tumors will be included:

Benign tumors

1. Nephroblastomatosis

2. Metanephric adenoma

3. Multilocular cystic nephroma

4. Angiomyolipoma

Malignant tumors

1. Wilms tumor

2. Renal cell carcinoma

3. Clear cell sarcoma

4. Mesoblastic nephroma

